# Early Metabolomic Markers of Acute Low-Dose Exposure to Uranium in Rats

**DOI:** 10.3390/metabo12050421

**Published:** 2022-05-07

**Authors:** Stéphane Grison, Baninia Habchi, Céline Gloaguen, Dimitri Kereselidze, Christelle Elie, Jean-Charles Martin, Maâmar Souidi

**Affiliations:** 1 Institut de Radioprotection et de Sûreté Nucléaire, PSE-SANTE, 92262 Fontenay-aux-Roses, France; celine.gloaguen@irsn.fr (C.G.); dimitri.kereselidze@universite-paris-saclay.fr (D.K.); christelle.elie@irsn.fr (C.E.); maamar.souidi@irsn.fr (M.S.); 2 C2VN, INRAE, INSERM, Aix Marseille University, 13007 Marseille, France; baninia.habchi@inrs.fr (B.H.); jean-charles.martin@univ-amu.fr (J.-C.M.)

**Keywords:** uranium, low dose, acute, contamination, metabolomics, diagnostic, N-methyl-nicotinamide, tryptophan

## Abstract

Changes in metabolomics over time were studied in rats to identify early biomarkers and highlight the main metabolic pathways that are significantly altered in the period immediately following acute low-dose uranium exposure. A dose response relationship study was established from urine and plasma samples collected periodically over 9 months after the exposure of young adult male rats to uranyl nitrate. LC-MS and biostatistical analysis were used to identify early discriminant metabolites. As expected, low doses of uranium lead to time-based non-toxic biological effects, which can be used to identify early and delayed markers of exposure in both urine and plasma samples. A combination of surrogate markers for uranium exposure was validated from the most discriminant early markers for making effective predictions. N-methyl-nicotinamide, kynurenic acid, serotonin, tryptophan, tryptamine, and indole acetic acid associated with the nicotinate–nicotinamide and tryptophan pathway seem to be one of the main biological targets, as shown previously for chronic contaminations and completed, among others, by betaine metabolism. This study can be considered as a proof of concept for the relevance of metabolomics in the field of low-dose internal contamination by uranium, for the development of predictive diagnostic tests usable for radiotoxicological monitoring.

## 1. Introduction

Anthropization produces pollution and threatens the health of the population [1]. For example, the operation of nuclear facilities (mines and mills) using uranium has become a health concern as, although uranium occurs naturally in the Earth’s crust [2,3], it can be released into the environment in larger quantities around mining sites, in war zones, or during industrial incidents/accidents [4,5,6,7,8]. In addition, natural uranium (NU) is a radioactive heavy metal whose dual toxicity (chemical and radioactive) depends on its isotopic form. Depleted and natural forms of uranium are more chemically toxic than enriched forms of U-235, whereas the latter are more radiologically toxic [9,10]. Nevertheless, although its toxicity seems to be well characterized at high doses, current scientific knowledge does not allow us to rule out the risk associated with low-dose exposure to NU potentially faced by populations and nuclear workers [11,12]. In fact, neither epidemiological studies, which are too uncertain, nor the tests used in clinical analysis, can be used to estimate the risk of adverse effects at these low levels of exposure to NU. Occupational uranium contamination is currently monitored by checking the daily radiochemical profiles of urine and feces and additionally measuring clinical chemistry parameters to diagnose any kidney damage when appropriate [13]. However, this may be insufficient in the low-dose range of exposure (considered non-toxic) as these tests are designed to identify acute toxicity and are not relevant for the detection of delayed morbidity [14]. In order to estimate such a risk and to refine radiation protection and the medical monitoring of workers, it is first necessary to establish a relationship between biological signs and adverse physiological effects [15]. Using analytical techniques that are sufficiently sensitive and specific to discern an abnormal biological signal in the homeostatic background could be relevant for this purpose when identifying biological markers for low-dose NU exposure [16,17,18].

Thus, in the field of low-dose chronic contamination by NU, experimental studies conducted in rats have shown that metabolomics could effectively be used to detect the effects of low contamination [19] and reveal a non-linear dose effect combined with a time relationship [20]. Among the most discriminant features highlighted (which could be used to help to develop a diagnostic exposure test), the level of N-methyl nicotinamide decreased in urine metabolome in contaminated rats, as previously observed with high doses [21]. Nevertheless, it is also relevant to consider the acute exposure that can prevail with occupational accidents and that the biological effects of chronic exposure may differ from those of acute exposure [22,23]. Therefore, in order to define the biological kinetics of acute exposure to low doses of NU, several study periods must be defined for the purposes of identifying early, late, or permanent biomarkers that could be used in clinical follow-up for accidentally contaminated workers. Nevertheless, the identification of early markers of acute exposure seems relevant when focusing on the early period of the biological response to identify the most sensitive metabolisms and better understand the adverse effects of uranium.

**Figure 1:**
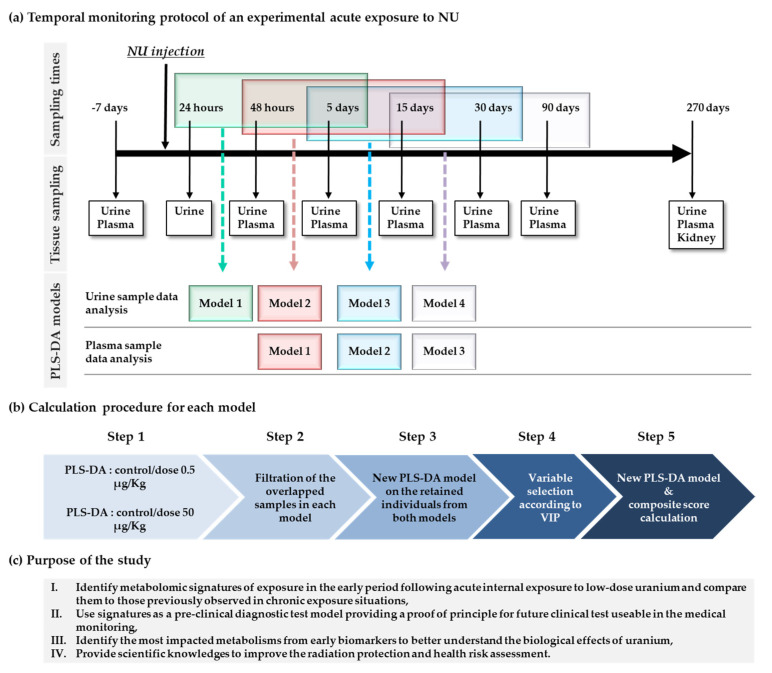
(**a**) Urine and plasma sampling times after acute exposure to NU and sampling time models defined for Partial Least-Squares Discriminant Analysis (PLS-DA). (**b**) Data processing pipeline patterns. (**c**) The main objectives of this study.

In this context, this present study is based on time-course design in an in vivo rat model (Figure 1) where urine and plasma samples were collected periodically over 9 months after the exposure of young adult male rats to uranyl nitrate. The aims are to (i) investigate metabolomic signatures that are significantly altered in the first period after NU exposure, (ii) use it as a potential diagnostic test, (iii) highlight the most affected metabolisms between these early biomarkers to better understand the impact of acute exposure to low doses of NU, and (iv) provide new insights that can be used in radiation protection and health risk assessments [24].

## 2. Results

### 2.1. Clinical Monitoring of Animals

Total body and kidney weights showed no significant statistical differences between the control and contaminated groups at euthanasia (Table 1). Nevertheless, statistically significant dose-dependent concentrations of NU in kidneys can be detected 270 days after exposure. According to chemical parameters, statistically significant differences can be seen exclusively for the higher-administrated dose of NU (500 µg/kg) for magnesium, phosphorus, total proteins, and uric acid 5 days after exposure. These dysregulations were associated with a ×1.7 increase in the 24 h urinary volume on day 5, which can be associated with kidney failure. No similar effects can be seen 9 months later except for a slight increase in uric acid, also exclusively shown at the higher dose of NU. This shows, however, that the minor early kidney failure suffered at the highest administered dose of uranium seems to persist in the long term, but less intensely, indicating partial recovery and reversible kidney failure.

### 2.2. Metabolic Profile Analysis of Mass Data Features

The first goal was to compare the previous results obtained in chronic exposure conditions with those obtained under acute exposure using the same analytical (C18 chromatographic column) and biostatistical procedure [20].

#### 2.2.1. Change in Urinary Metabolic Profiles over Time

When monitoring acute exposure on a long-term basis, it appears necessary to estimate the effect of aging on metabolomic profiles compared to low-dose ionizing radiation as a possible confounding factor.

**Figure 2:**
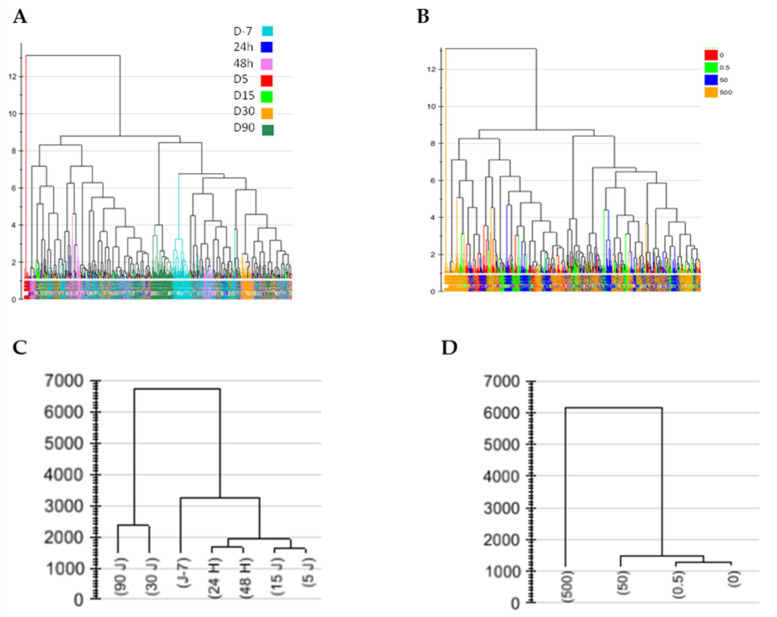
Hierarchical clustering of observations based on the 30 principal component analyses (**A**,**B**) or 10 (**C**) or 20 (**D**) PLS-DA components performed on all urine sampling times and NU doses. Each component influence is weighted according to its respective eigenvalue. Hierarchical classification analysis with Ward as the clustering method and tree sorted by size. In (**A**), sample colors correspond to sampling times; in (**B**), sample colors refer to NU doses. (**C**,**D**) represent the c(corr) vector, showing how well the X variables (mass features intensity) fit with the Y variables (either time factor in (**C**) or the dose factor in (**D**)). This vector can be seen as the barycenter of each time group or NU dose group. In (**C**), the time response was highly significant in PLS-DA analysis (P after cross-validation ANOVA = 0), whereas in (**D**), the dose response was not (P after cross-validation ANOVA = 1).

A strong sampling time effect was demonstrated by the PCA (Figure S1a) and the PLS-DA with sampling time as the Y predictor variable confirmed this difference (CV-ANOVA *p* = 0, R2Y(cum) = 85.2%, Q2(cum) = 67.8%, data not shown). Significant drift occurred after 30 days. However, no clear dose effect was observed with PCA (Figure S1b) or when the PLSDA model was calculated at all times and doses with the dose effect as the Y dummy matrix (data not shown)**.** This result can be explained by the strong variation in contamination due to varying NU intraperitoneal injections (IP). Therefore, the data processing procedure was applied to refine the analysis. Either the PCA or the PLS-DA analyses indicated that the effect of time/aging was much superior to that of NU dose contamination (Figure S1). In this instance, the time effect was more contrasting from day 30 and beyond (Figure 2).

The results obtained from the PLS-DA of mass features show that there is no discriminant variable common to all sampling time models (Supplementary Materials, Tables S1 and S2). However, a total of 19 discriminant variables in common between at least two of the calculated models were selected and are predictive of uranium exposure mainly between 24 h and 30 days (e.g., Q2 > 0.5, Table S2).

Based on these results, contamination biomarkers apparently exist for the early period and others may potentially be more relevant for the late period. Therefore, to identify these specific early biomarkers for the longest period following acute exposure regardless of the time collection point, discriminant variables common to consecutive periods, especially between model 1 and model 2 (i.e., early period between 24 h and 15 days, Table S1), were used to detect nine common discriminant variables. Control and low-dose contaminated rats were clearly differentiated in the new reduced PLS-DA model, from which a composite score was calculated and validated using the Area Under the Receiver Operating Characteristics curve (AUC-ROC = 1, Table 2). Two interesting biomarkers were detected from these nine discriminant variables, M137T39 and M184T138. These two variables were identified as the [M+H]^+^ ions of 1-methylnicotinamide (0.05 ppm) and 4-pyridoxate (0.5 ppm), respectively, based on the accurate m/z and retention time value and on the similar fragmentation patterns obtained for a protonated compound 10^10^ from urine samples and the authentic standard solution (Supplementary Materials, Figure S2).

We then tentatively tried to confirm in a validation step if this composite score is valid in the high-dose contamination range. The same procedure (based on these nine discriminant variables characteristic of low NU doses before 15 days) was applied to control and contaminated groups with the highest dose (500 µg/kg). In this case, the calculated model effectively predicted the first three models run between 24 h and 30 days, whereas it was not possible to discriminate between non-contaminated and contaminated rats after 30 days (Q2(cum) < 30%) (data not shown).

#### 2.2.2. Time Effect in Plasma Profiles

In the same way as urine, with plasma samples, the PCA performed with all samples (all sampling times for exposed and non-exposed rats) was not able to differentiate between the different NU doses, whereas all sampling times could be differentiated with the third principal component (data not shown). However, if a time effect was confirmed by PLS-DA in plasma metabolome, no dose effect was observed (Figure S3).

As was the case for urine, no common discriminant variables for all sampling time models (Table S3) were detected.

Therefore, the variables in common to at least two models were selected, and new models were calculated using the 27 common variables (Table S4). The PLS-DA models and the composite score (AUC-ROC > 0.8) clearly differentiate between controls and low NU doses up to 30 days, whereas after 30 days, the 27 selected biomarkers are not able to differentiate between contaminated and non-contaminated rats (Table S4).

#### 2.2.3. Long-Term Kidney Profile after Low-Dose Exposure

In order to highlight a possible residual metabolic effect on the kidneys, a metabolomic analysis was performed on renal tissue 9 months after exposure. Nine months after NU contamination, the rat’s kidney profiles entered in the PLS-DA model were calculated with NU contamination as the predictor variable Y in the matrix, including all NU doses and control samples at 9 months. NU-contaminated rats could not be differentiated. In addition, no differentiation between the controls and the different doses of NU was validated even after selecting the variables (data not shown).

### 2.3. Metabolic Profile Analysis of Annotated Data Matrixes

Data obtained from C18 and hydrophilic interaction chromatography (HILIC) chromatographic analyses and both ionization modes were annotated and merged into a single dataset fully annotated using our in-house database as described in the methods section.

#### Discrimination of Rats Contaminated with Low Doses of NU as a Function of Time in the Concatenated Urine and Plasma Annotated Data Tables

The same procedure was applied separately to the annotated urinary and plasma data matrixes (Tables S5 and S6). The results obtained from each biological matrix are summarized in the Supplementary Materials, because more worthwhile results were obtained from the application of the same procedure to the annotated concatenated urine and plasma data. As observed above for C18 positive ionization urinary and plasma data, sampling times were clearly differentiated with PCA. However, contaminated rats could not be clearly differentiated from non-contaminated rats (Figure S4).

The same calculation processing procedure for the PLS-DA model was applied to these concatenated urine and plasma data between control group and contaminated NU doses (0.5 and 50 µg/kg) for the three different successive sampling times (Table S7). Metabolites that are common to the different successive time groups were then selected, and new PLS-DA models were calculated based on these common metabolites. Six discriminating variables were detected in common between the different time models (Table 3). The PLS-DA calculated on the three models (from 48 h to 90 days) using these six common discriminant variables was not validated (R2Y(cum) = 26.6%; Q2(cum) = 24.4%; *p*-value = 3.04766 × 10^−15^). However, the 1-methylnicotinamide metabolite is one of these six common discriminant variables. These results demonstrate that the metabolites associated with uranium exposure are not the same over time following acute exposure and different combinations of biomarkers are more specific to each period.

However, 36 discriminant variables are in common to at least two models (Table 3 and Table S8 for more details). The PLS-DA models calculated from these metabolites effectively predicted the rat status between 48 h and 30 days (R2Y(cum) = 55.3%; Q2(cum) = 48.5%; *p*-value = 2.89677 × 10^−24^) and showed good permutation tests. However, the contamination prediction appeared less robust after 30 days (Q2(cum) < 38%). Beyond 30 days, other metabolites specific to the later period (between 15 to 90 days) are needed to differentiate between the control and contaminated groups (Table 3 and Table S8).

## 3. Discussion

While studies using targeted [25,26] and non-targeted [27] analyses have looked at the effects of high- and low-dose chronic exposure to uranium [12,28,29] to assess health risks [30], the effects of acute low-dose exposure to uranium are still poorly described, even though they are a part of the occupational exposure faced by nuclear workers. To fill this gap, identifying biological signatures that could be used to diagnose the sub-toxic effects of low-dose internal exposure would be relevant to improving the radiation protection and medical monitoring of nuclear workers. Thus, in this present study, variations in dose effects over time were monitored by metabolomic analysis to detect specific biomarkers of acute low-dose exposure to NU.

Regarding zoometric and clinical monitoring, total body and kidney weights did not differ between the control and NU-contaminated groups of animals. Nonetheless, for the highest dose used (500 µg/kg), the statistically significant deregulation observed in urine for renal chemical markers (magnesium, phosphorus, total proteins, and uric acid) and the increase in 24 h urine volume reveals its toxicity and exclude it from the low-dose range. Thus, this dose can be considered as a high dose for which renal failure was observable between 5 days after contamination to 9 months. This is highlighted by the detection of a slight increase in urinary uric acid associated with a significant increase in the NU concentration measured in kidneys (Table 1). Renal NU retention seems to increase with the dose and persist for a long time after exposure. Unsurprisingly, kidneys and bones are known to be biological uranium reservoirs. Progressive release through bone turnover can thus partially explain this long retention time observed in kidneys [31]. Furthermore, given that xenobiotic toxico dynamics are closely related to toxicokinetics, the longer the NU is stored, the more cytotoxic it will become in the kidney [32,33]. Nonetheless, if this high dose can induce lasting kidney damage, it also appears that kidney injuries seem to decrease over time, indicating recovery. For the lower two doses used, residual NU concentrations and measured levels of urinary clinical markers are close to the values measured in control animals. This confirms the absence of renal failure and indicates that these doses are in the low-dose range. To assess the underlying biological effects of such a low level of exposure, a highly sensitive analytical approach, such as metabolomics, can be used to measure molecular dysregulations that could act as predictive markers of renal sensitivity or even risk indicators for subsequent morbidity [34].

With this aim, it seems relevant to first compare the effects of acute exposure with those previously observed in chronic exposure to uranium, by using the same Data Processing Pipeline for which only the most discriminant signals were chemically identified from the LC–MS signals measured [20].

As shown previously with chronic contamination [20], a significant effect of aging on metabolism is observable. It seems necessary to estimate the impact of age as a confounding factor [16,35] in order to identify specific early biomarkers for low-dose NU exposure or reduce the time window of the analyses. As expected in this present study, the effect of aging is clearly superior to that of the dose–effect relationship measurable in the urinary and plasma metabolomes. Indeed, by combining all sampling times in one single statistical analysis, a significant time effect was observable in the PCA and PLSDA (Figures S1a and S3a) for both urine and plasma samples. A dose–effect relationship could not be revealed (Figures S1b, S3 and S4) and no common discriminant variable was identified between all times models (Tables S1 and S2). Here, too, the effect of aging is more important than the effect of acute low-dose exposure to NU and no constant metabolic pattern signifying contamination was observable. Aging is an endogenous factor that interferes with the effects of low doses of NU and must be taken into account [35].

According to initial results on the effects of NU, the absence of metabolomic perturbation observable 9 months after exposure in renal tissue may suggest the absence of long-term sequelae. Moreover, in the absence of morbidity, the changes in effects observed could be explained by a homeostatic return a few weeks after contamination. Nevertheless, biomarkers of contamination for the early period and others signifying the late period can be detected in both urine and plasma by dividing the temporal data into different study models. A statistical score that can be used as a diagnostic test for uranium exposure can also be calculated [36] from the respective concentrations of these metabolomic markers for low-dose range exposure, thus compensating for the lack of sensitivity of clinical tests.

Early effects are observable in urine samples by PCA for the first 30 days after exposure (relatively unaffected by aging as a clear time effect is observed after 30 days) and then change between 30 and 90 days. Nine common discriminant features exist between the first two sampling models run between 24 h and 15 days (Figure 1). The composite score calculated from these models clearly differentiated between 24 h and 30 days at a low dose level and was efficient beyond this level for the group exposed to the high dose (500 µg/kg). In addition, this score can be used for longer, up to 30 days, at high doses (instead of 15 days for low doses), while 19 features common to at least two models are required at low doses to diagnose animals exposed during the first 30 days. At low doses, few features appear necessary to identify exposed and unexposed animals during the first 15 days after exposure, when the effects of NU are certainly most intense. Conversely, over a longer period, a greater number of markers will be needed to predict a diminishing biological effect. It should also be noted that this dose–response relationship can be extended over time (up to 30 days) when the dose of NU is higher (500 µg/kg). This close relationship between dose and time suggests that these nine features may be surrogate markers for exposure, as previously shown in a study of chronic NU exposure, where effects appeared earlier as the dose increased [20]. Due to these temporal dose–response relationships, these diagnostic scores could also reflect the severity of exposure. As one of the main objectives of this study was to compare the effects of this acute NU exposure with those of chronic exposure, it is interesting to note that 1-methylnicotinamide (NMN), already identified as deregulated in urine by chronic NU contamination [20], was detected among these nine early markers of acute NU exposure. According to current knowledge and experimental observations, NMN increases in the renal cortex and plasma and decreases in the urine of rats exposed to a high toxic dose of uranium [21,37], also associated with the effects of chronic low-dose exposure in urine and renal tissue [27]. Others have also identified NMN in the salivary analyses of Kuwaiti adolescents living in an area contaminated by uranium during childhood [38]. Thus, these observations suggest that NMN metabolism could be impacted by uranium exposure and, in addition, could be a marker for renal sensitivity through membrane transporter interaction (OCT2) in tubular cells [21,39,40]. NMN is also a metabolite of niacin, the B3 vitamin. This raises the hypothesis of therapeutic treatment for NU contamination and certainly deserves an evaluation using NU-treated rats.

In addition to urinary analysis, plasma analysis could offer a more complementary view. As in urine, discriminating variables related to time have been detected in plasma samples. The statistical model calculated using the common discriminant variable for the first sampling periods is predictive up to 30 days and shows that these discriminant variables vary, especially before and after 30 days, where a break in metabolomic profiles is clearly observed. Unlike urine, no biomarker linked to the nicotinate–nicotinamide pathway could be detected in the plasma. Nevertheless, the validation of the discriminant profile obtained in plasma using the ROC curve is robust and, as with urine, can be used to establish a reliable diagnostic test model, at least during the first month after acute contamination. Finally, to deepen clinical observations made 9 months after exposure for both urine and plasma samples, a metabolomic analysis was run based on renal tissue biopsies and showed that under our experimental conditions, it was not possible to differentiate between contaminated and non-contaminated animals. Thus, this result seems to confirm the clinical analysis that low doses of uranium do not cause lasting biological damage for kidneys.

In a second step, the objective is to further this study with a targeted analysis of signals exclusively identifiable by spectral databases to initially eliminate spurious signals that may be sources of redundancy and confusion in the statistical analysis [41]. This methodological approach should make it easier to interpret results by focusing exclusively on a large dataset of fully identified metabolites.

From the pre-annotated and combined data matrixes, a time/aging effect much larger than the treatment effect (NU) is again observable by PCA. This is demonstrated by the lack of general differentiation between contaminated and non-contaminated rats. However, PLS analysis identified six discriminant variables common to the different temporal patterns analyzed between 48 h and 30 days after exposure (Table 3). Among these variables, 1-methylnicotinamide (NMN) was systematically identified and associated with a decrease in its urinary clearance in contaminated animals. This result seems to confirm its ability to act as an endogenous biomarker sensitive to NU.

Furthermore, a set of 36 discriminating metabolites detected in at least two temporal models was selected and the new statistical model calculated using this set effectively predicts outcomes between 48 h and 30 days. This result thus confirms the existence of early NU biomarkers (before 30 days post exposure) and later biomarkers (Table 3, Tables S7 and S8). Regarding these 36 discriminating metabolites, it can be assumed that different metabolic pathways could be affected by uranium. The results showed that indoleacetic acid and tryptamine may be related to the tryptophan metabolism, which is also related to the nicotinate–nicotinamide pathway by quinolinic acid and 1-methylnicotinamide, as targeted by the early NU markers, as observed after chronic exposure [27].

**Figure 3:**
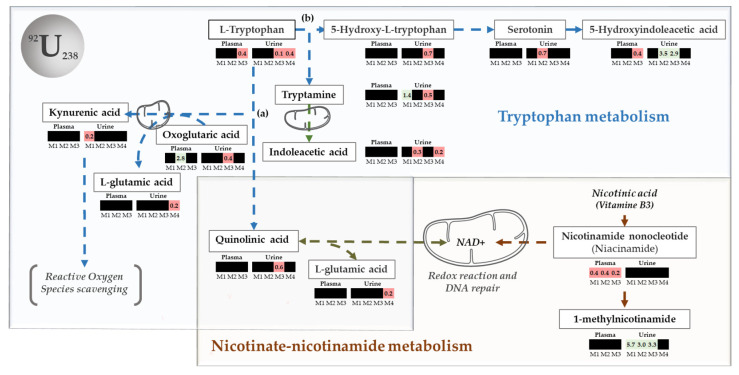
Main pathways targeted by NU at low doses: The main discriminating metabolites identified in urine and plasma are in white boxes. They show that tryptophan and nicotinate–nicotinamide metabolism (https://smpdb.ca; 2021) represent the main targets of acute NU exposure: (**a**) the kynurenine pathway leading to nicotinate–nicotinamide metabolism and NAD+ synthesis and (**b**) the serotonin pathway are both highlighted by the metabolomic analysis of urine and plasma samples collected at different times following the exposure of rats. The heat maps associated with each discriminating metabolite show both their relative levels change (green decrease and red increase) and the corresponding time for it was identified as the most discriminating (black if not significantly discriminating).

Interestingly, nicotinate–nicotinamide metabolism (related to 1-methyl-nicotinamide) has also been identified as deregulated by other sources of oxidative stress, such as gamma rays [42]. Tryptophan metabolism is also involved in inflammatory processes [43] and in several diseases, including chronic kidney disease [44]. Three main pathways arise from tryptophan metabolism: the aryl hydrocarbon receptor (AhR) signaling pathway and the kynurenine (KP) pathway that are involved in the immune response [45,46] and, finally, the serotonin pathway [47]. The AhR pathway, which may have an anti-inflammatory effect by regulating signaling cytokines processes [46], seems to be particularly affected by the dysregulation of L-tryptophan, tryptamine, and indole acetic acid. Indoleacetic acid is also a by-product of 5-hydrotryptamine (serotonin), whose pathway has been identified as a target of uranium [48] (Figure 3b). The kynurenine pathway, which leads to nicotinamide adenine dinucleotide (NAD+) via quinolinic acid, is also deregulated by uranium and linked to nicotinate–nicotinamide metabolism (already known to be affected by uranium) (Figure 3a).

Other discriminant metabolites, such as betaine, which is an amino acid derivative, may have possible anti-inflammatory, antioxidant [49,50], and anticarcinogenic effects [51]. In addition, dimethylglycine is a metabolite involved in betaine metabolism, similar to dimethylglycine, and has also been associated with normal kidney function. The betaine metabolism here marked by betaine and dimethylglycine has been also associated with a normal kidney function [52]. Note that its antioxidant effects may reduce genotoxicity, as observed in the liver, by the effect of ionizing radiation [53,54] and help tolerate oxidative stress of heavy metals [55]. Finally, glycine and betaines are renal osmolytes that can be increased in urine (as observed in this study) in the case of renal disease and diabetes [56].

In addition, hypotaurine may be associated with inflammation through its antioxidant role and involvement in heavy metal detoxification processes [57,58,59,60,61] and may also have a wider impact on taurine metabolism [62], which appears to have a beneficial role in renal disorders [63]. Other discriminating metabolites, such as gluconolactone and saccharate, are glucuronic acid derivatives involved in the glucuronidation of toxic compounds. They are also involved in the phase II detoxification process of many endobiotics or xenobiotics. Finally, amino acid metabolism that can be related to betaine, dimethylglycine, phenylalanine, L-phenylalanine, L-pipecolic acid, beta-alanine, beta-alanine, L-proline), carbohydrates (pentose), the citric acid cycle (L-malic acid), and allantoin may also be associated with an inflammatory state or renal disorders, such as chronic renal failure [64,65,66].

All these results reveal the existence of different metabolic profiles that could be associated with variations in effects over time. These molecular profiles could also be used to calculate score values, which could be used as a diagnostic test for the effects of accidental exposure to low doses of NU. However, it is once again noted that composite biomarkers designed for short-term use (<30 days) cannot be effective over a long period of time because the panels of bioindicators indicated shift over time. Thus, for more extensive follow-up, it would be necessary to use sets of biomarkers specific to the short term and others for the long term.

This alternative method can also be used to diagnose contaminated animals over a 30-day period. As NU effects appear to decrease progressively in intensity over time, 53 additional variables were necessary to construct a discriminative, significant, and predictive model (Model 3, Table S7). Nevertheless, when the dose of NU is higher (500 µg/kg), persistent effects can be observed beyond 15 days, up to 30 days after exposure. This result suggests that the persistence of the effect is highly dependent on the dose and that the early metabolomic profile observed could be highly specific to NU exposure. Similarly, the diagnostic test defined at low doses becomes more accurate over a longer period of time, in proportion to the dose received. Plasma and urinary matrixes were then analyzed separately (Tables S5 and S6) to obtain a view that relates more and directly to biological compartments. A total of 3 of the 95 most discriminating urinary metabolites distributed across the different time sampling models are related to nicotinate–nicotinatide metabolism and 8 to tryptophan metabolism (Table S5). The impact of uranium is observable for 90 days after exposure. In plasma, 60 discriminating variables were identified, including nicotinamide, which is also a part of the nicotinate–nicotinamide pathway, and other metabolites related to tryptophan metabolism (Table S6). Interestingly, this approach reveals serotonin deregulation in urine that was not revealed by the combined matrix analysis. A previous study had indeed shown that the serotonin pathway could be affected by NU [48], although this was not clear [67]. Thus, while the global analysis including both plasma and urine summarizes the main information on observable effects, the separate and specific analysis of biological compartments allows for a more in-depth observation of the mechanistic processes that may be involved in a biological effect while providing a more comprehensive view of all deregulated metabolites (Figure 3). Similarly, despite a significant time effect, the discriminating models obtained, combined with excellent validation scores for the ROC curve, confirm that it is also possible to establish diagnostic tests from urine or plasma samples exclusively using this method.

To conclude this section, targeted analysis of annotated data appears to be more attractive for mechanistic studies than direct analysis of mass signals [68] due to the limited coverage of metabolites referenced in spectral databases and the fact that the risk of interference from unidentified ionization adducts may also be significant at the risk of degrading the statistical analysis of the data.

In addition, the targeted analysis of the annotated arrays confirmed the previous results for the nicotinate–nicotinamide pathway and reinforced those obtained for the tryptophan pathway [27].

## 4. Materials and Methods

### 4.1. Materials

Ultra-pure water from a Milli-Q^®^ system (Merck Millipore, Guyancourt, France) was used. Acetonitrile and formic acid of the highest commercial grade were obtained from Sigma Aldrich Chemicals (Fontenay-sous-Bois, France). Natural uranium (NU, Mc Arthur) was obtained from CERCA (Pierrelatte, France). Uranyl nitrate hexahydrate (UO_2_(NO_3_)2·6H_2_O) was prepared in a saline solution (NaCl) to obtain three different concentrations of uranium (0.5; 50; and 500 µg/kg).

### 4.2. Animal Treatment and Sample Collection

In all, 80 Outbred Sprague–Dawley rats (8 weeks old) were obtained from Charles River Laboratories (L’Arbresle, France). They were housed and maintained in a monitored environment (temperature 21 °C and 50% humidity) under a reversed light–dark cycle (dark from 20:00 p.m. to 08:00 a.m.). The rats were split into 4 groups (20 rats/group) and exposed by the intraperitoneal injection of NU at the concentrations of 0.5, 50, and 500 µg/kg for the three contaminated groups. The control group was injected with a saline (NaCl) solution. Urine and plasma samples were collected periodically throughout the experimental protocol at six and seven sampling times, respectively, for metabolomic analysis. Sampling intervals were shortened during early post-exposure times to focus on the early biological effects of uranium. In addition, to verify the long-term absence of any risk of renal impairment potentially caused by such exposure and thus confirm that the lowest doses used in this study are non-toxic, urine and plasma were collected at 9 months (270 days) for clinical monitoring. All animals were euthanized at 9 months old, and kidney samples were stored at −80 °C prior to analyses. The experimental procedures were approved by the Animal Care Committee of the Institute of Radioprotection and Nuclear Safety (IRSN) and complied with the French regulations for animal experimentation (Ministry of Agriculture Act No. 87-848, 19 October 1987, modified 20 May 2001).

### 4.3. Clinical Monitoring

#### 4.3.1. Animal Monitoring

Throughout the experimental protocol, the physical condition of each animal was monitored daily, water consumption weekly, and body weight gain at each sampling time during the early time after contamination by NU (24 h to 30 days) and monthly beyond to ensure good health and housing conditions. At euthanasia, kidney weights were also measured to detect any pathological features of the kidneys.

#### 4.3.2. Chemical Monitoring in Urine and Plasma Samples

Biochemical measurements of thawed urine and plasma samples were taken with an automated spectrometric system (Konelab 20 from Thermo Electron Corporation, Cergy-Pontoise, France) and the manufacturer’s biological chemistry reagents and protocols. For the purposes of diagnosing kidney failure, some biochemical and clinical parameters were measured in urine, including volume/24 h, albumin, chlorine, creatinine, glucose, magnesium, potassium, sodium, total proteins, urea, and uric acid. Creatinine and urea were measured in plasma. Creatinine clearance was calculated to estimate the glomerular filtration rate. Biochemical and clinical parameters are reported as the means ± the standard error of the mean (SEM). Statistics were performed with SigmaStat statistical software (SPSS, Paris, France) to calculate items such as Student’s *t*-test in normal populations or the rank sum test in non-normal populations in order to compare the control and contaminated groups. Statistical significance was defined by a *p*-value less than 0.05.

#### 4.3.3. Uranium Level in Urine and Kidney Samples

To measure NU burden in kidneys and urine, kidney samples were prepared by adding 8 mL of ultrapure nitric acid (69%) and 2 mL of hydrogen peroxide (30%) and then mineralized in a 1000 W microwave (Ethos Touch, Milestone Microwave Laboratory Systems, Sorisole, Italy) heating at a rate of 9 degrees per minute up to a temperature of 180 °C, which was then maintained for 10 min. The urine samples were only diluted before measurements. Samples were analyzed by ICP-MS (XSERIES 2, ThermoElectron, Villebon-sur-Yvette, France). Experimental conditions were optimized by using a multi-element standard solution (Thermo Electron, Villebon-sur-Yvette, France), and bismuth 209 was added to all samples as an internal standard at 1 µg L^−1^. A calibration curve was calculated based on a standard solution of uranium (Spex, Horiba Jobin Yvon, Longjumeau, France) at 1000 mg L^−1^ in 2% nitric acid freshly diluted to obtain 0, 0.001, 0.005, 0.01, 0.1, 0.5, and 1 µg L^−1^ in 2% nitric acid. A linear relation–count number (^i^U) = f([^i^U]) was calculated for each isotope, i = [235;238] with [^i^U] equal to the isotope concentration in µg L^−1^. The ICP-MS limit of detection for uranium is 1 ng L^−1^.

### 4.4. Metabolomics Analysis

In order to minimize the analytical variability inherent in preparation and analysis, all group samples were first randomized, prepared, and analyzed in different analytical batches (80 to 100 samples by batch).

#### 4.4.1. Sample Preparation

a. Urine samples

Urine samples were placed in a centrifuge for 15 min at 11,000 rpm and 4 °C, and the supernatant was diluted with ultrapure water (1:4 *v*/*v*). The samples were again placed in the centrifuge for 20 min at 11,000 rpm and 4 °C, and 30 µL of the supernatant was transferred into HPLC vials and stored at −80 °C prior to analysis.

b. Plasma samples

Plasma samples (40 µL) were diluted and homogenized with 400 µL of cold methanol (−20 °C), followed by incubation for 30 min at −20 °C to precipitate proteins. The samples were then placed in a centrifuge for 15 min at 11,000 rpm and 4 °C. Plasma supernatant was collected in a centrifugal filter (VWR^®^, Rosny-sous-Bois, France, 10 KDa) and centrifuged a second time for 45 min as previously. The supernatant was dried under a stream of nitrogen and stored at −80 °C until analysis. Dried plasma extracts were dissolved in 100 µL of water/acetonitrile (90/10, *v*/*v*). Samples were vortexed for 1 min and placed in a centrifuge for 15 min at 11,000 rpm and 4 °C. A total of 30 µL of the supernatant was transferred into HPLC vials and stored at −80 °C prior to analysis.

c. Kidney samples

An amount of 20 mg of kidney tissue was added to 600 µL of prechilled methanol and homogenized for 5 min at 25 Hz in a mixer mill powerful grinding system (MM400, Retsch Technology, Éragny, France). Samples were incubated for 30 min at −20 °C and centrifuged for 15 min at 11,000 rpm and 4 °C. The supernatant was collected in centrifugal filters (VWR^®^, France, 10 K) and centrifuged a second time for 45 min and dried under nitrogen flux. The dry residue was re-dissolved in 200 µL of water/acetonitrile (50/50, *v*/*v*) and centrifuged as previously. Finally, the supernatant was transferred into HPLC vials and stored at −80 °C prior to analysis.

d. Quality control (QC) and blanks

A quality control sample (QC, a pool of all samples of a biological matrix) was injected several times at the beginning of each batch for column equilibration and interspersed every set of 5 samples throughout the experimental sequence to evaluate the data quality (repeatability, drift correction). Some initiatives recommend the use of internal standard in the samples as a more robust procedure to monitor the performance of the analytical system and peak alignments [69]. However, there is yet no formal recommendations on how and what internal standards to be used in metabolomic experiments, compared to the use of QC samples, for instance. MS/MS experiments were also performed on the QC sample at the end of the sequence to improve metabolite annotation by comparing the MS/MS spectra with our in-house database, comprising over 800 metabolite standards. In addition, an extraction blank of the solvent mixture was prepared at the same time as the biological samples. The extraction blank was analyzed at the beginning of the experimental batch sequence.

#### 4.4.2. Liquid Chromatography Mass Spectrometry Analysis

All samples were analyzed using an Orbitrap (Q-Exactive Plus, Thermo Fisher Scientific San Jose, CA, USA) hyphenated to a high-performance liquid chromatography system, Surveyor LC (Thermo Fisher Scientific). LC–MS experiments were acquired in polarity switching mode in the *m*/*z* 80–1000 range with a mass resolving power of 35,000 full width at half maximum (FWHM). The following ESI conditions were applied: electrospray voltage of 3.5 kV, an S-lens RF level of 55, and a capillary temperature of 320 °C. The sheath gas flow (nitrogen) was set at 30 arbitrary units (a.u.). Sheath gas, auxiliary gas, and sweep gas flow rates were maintained at 30, 8, and 0 arbitrary units (a.u.), respectively.

Chromatographic separation was performed using two column types to enlarge the metabolome coverage. For reversed phase (RP) chromatography, a Hypersil Gold C18, 100 × 2.1 mm × 1.9 um (Thermo Fisher Scientific, Illkirch, France) was used with a mobile phase consisting of water plus 0.1% formic acid (A) and acetonitrile plus 0.1% formic acid (B) at a flow rate of 0.4 mL/min and a column temperature of 40 °C. The gradient started at 0% (B) for 1 min, decreased to 100% (B) over 10 min, was maintained at 100% B for 2 min, and then returned to 0% (B) in 1 min and equilibrated the column at 0% (B) for 2 min (16 min in total). For hydrophilic interaction liquid chromatography (HILIC), a Sequant zicHILIC 5u, 200A, 150 × 2.1 (Merck, France) column was used at a flow rate of 0.25 mL/min and the mobile phase consisted of water plus 16 mM ammonium formate (A) and acetonitrile plus 0.1% formic acid (B) at a column temperature of 25 °C. The gradient started at 97% (B) for 2 min, decreased to 70% (B) over 8 min and to 10% (B) in 5 min, then was maintained at 10% (B) for 2 min, was returned to 97% (B) in 1 min and held at 97% (B) until the end of the gradient for column equilibration (27 min in total). The injection volume was 5 µL for both columns.

Tandem mass spectrometry (MS/MS) experiments were performed on the top five monoisotopic peaks using positive and negative ionization mode separately in the m/z 80–1000 range with a mass resolving power of 17,500 FWHM. The higher collision dissociation (HCD) condition was used with the following parameters: isolation width of precursor ions of 2 u, activation time of 50 ms, and normalized collision energy of 30% (arbitrary units).

#### 4.4.3. Data Pre-Processing and Statistical Analyses

a. Data pre-processing

Raw LC–MS data were converted from profile into the centroid mzXML file format. The data generated were pre-processed using XCMS script operated under R language (R version 3.4.0; 2018, The R Foundation for Statistical Computing, Vienna 2017, https://www.r-project.org). The XCMS procedure was applied in four main steps: peak picking, peak grouping, retention time correction, and a second peak grouping step. The *centWave* method and a non-linear LOESS alignment method [70] were used to extract peaks and for a retention time drift correction, respectively. For missing values, the peak filling method was applied to determine the intensity of the peaks from the raw data.

Once the raw data matrix was created, a filtration step was applied to remove variables with a poor extracted-ion chromatogram (EIC), a poor peak shape, and/or blank intensities greater than or equal to those found in data from the biological samples. In a second step, a normalization method was applied using a non-linear LOESS intensity correction based on the repeated injections of QC samples during the sequences in order to correct intra- and inter-batch drift. After this normalization phase, a second filtration step was applied by removing variables with relative standard deviation (RSD) in QC injections higher than 30%. Finally, a second data normalization phase was applied if needed (i.e., UV scaling or/and pareto scaling to enhance the relative importance of small peak intensities while keeping the data structure relatively intact).

Regarding metabolite annotation and the selection of discriminant variables, all detected ions were putatively annotated or identified thanks to an internal database of approximately 800 standards dedicated to each metabolomic analytical method and specific to each molecule, with the corresponding accurate *m*/*z*, RT, formula, and fragmentation spectrum. The experimentally accurate *m*/*z* and RT values were used to query the internal database with a mass error below 5 ppm and RT difference below 0.5 min using an automatic search tool freely available in the Workflow4Metabolomics collaborative portal [71]. To ensure the best metabolite annotations, we simultaneously considered the different proposals obtained for both columns and both ionization modes. If a metabolite was detected in different adduct forms in both ionization modes and/or both columns, the correlation coefficient was calculated between the corresponding adducts to confirm the annotation. In a second step, the different adduct intensities detected in the same ionization mode and the same column were summed and only one proposition was retained between the different columns and the ionization mode with the lowest RSD in the pool injections and/or the lowest mass measurement error. In addition, when the MS/MS spectrum was available (if the peak intensity for ions in the QC sample was high enough), it was compared with the standard MS/MS spectrum.

The most discriminant variables in a partial least squares discriminant analysis (PLS-DA) model were selected according to the variable importance in projection (VIP) of the PLS algorithm. Finally, metabolomic data were analyzed and discriminant variables were interpreted using Metaboanalyst 4.0 [72], SIMCA 17, and Excel 365 to calculate the composite score. The composite score is calculated with the NIPALS algorithm using the partial PLS correlation coefficients of relevant variables (metabolites) and combining them into an equation used to predict the class of new individuals [73,74,75].

b. Data processing

Multivariate statistical analyses were performed using SIMCA-P+ 12.0 software (Umetrics, Umeå, Sweden) and Metaboanalyst [76]. Principal component analysis (PCA) was applied after each pre-processing step to view data and detect outliers. PLS-DA models were validated by CV-ANOVA and a permutation procedure to check overfitting.

According to the rate of NU in urine measured at 24 h, the uranium contamination level varies significantly due to heterogeneous NU intraperitoneal (IP) injections. A pretreatment procedure based on data selection and cleaning was, therefore, applied using a supervised multivariate analysis to overcome this variation in contamination. The goal was to calculate a predictive equation (composite score) based on low doses (0.5 and 50 µg/kg) from the most clearly differentiated individuals in order to predict new samples. Groups of three sampling times were created in order to maintain enough individuals in each statistical model (Figure 1a). In step one, a PLS-DA model was calculated between the control group and each contaminated group (0.5 and 50 µg/kg) for each of the first three sampling times (Figure 1a,b). The calculated models were validated in most cases, but some control and contaminated samples were misclassified. The overlapped samples were filtered to improve final differentiation, then a new PLS-DA model was calculated for the individuals retained from both doses (i.e., dose 0.5 and 50 were grouped into a low-dose group). In addition, variables were selected according to VIP and a new validated PLS-DA model was generated and used to compute a composite score (Figure 1b). The same procedure was applied after shifting the time window to the next sampling step and so on, as showed in (Figure 1a). Each composite score was validated based on the receiver operating characteristic (ROC) curve calculated using Metaboanalyst. In order to identify biomarkers relevant for uranium contamination irrespective of sampling time, metabolites in common to at least two models were selected, and a new model was calculated. In step two, if the different successive time groups shared common metabolites (i.e., model 1 and model 2 in Supplementary Materials, Table S1), a reduced composite score was calculated and this equation was used to evaluate the effectiveness of these metabolites in predicting the individuals of model 1 and model 2 and then the possibility of predicting the individuals previously eliminated in the filtration steps.

This procedure was applied to the C18 positive ionization mode data matrix in order to compare the results with those previously published [20] under the same analytical conditions on chronic exposure data. Then, to improve biological data mining based on the metabolites identified, the same procedure was applied to the annotated metabolites from the four concatenated matrixes of each biological matrix (positive and negative ionization modes for HILIC and C18 columns).

Finally, in order to group discriminating data according to their involvement in metabolisms and biological functions, pathway enrichment was analyzed with the specific MetaoAnalyst module (https://www.metaboanalyst.ca/, 2019) using the Small Molecule Pathway Database (https://smpdb.ca/, 2019).

## 5. Conclusions

To conclude, although further analysis is required, the results obtained in this study show that different metabolic pathways can be affected by dose exposure to uranium at levels below renal failure. The metabolomic disrupts can mainly be observed on the nicotinate–nicotinamide pathway and tryptophan, which are already known to be involved in inflammatory processes. Other metabolites could be implicated in betaine, taurine, and glycine metabolisms, which are involved in antioxidant and detoxification processes, as well other. Carbohydrates, amino acids, allantoin, citric acid cycle, steroids, phospholipids, and fatty acids also appear to be possibly targeted by uranium and may contribute to biological mechanisms associated to an increase in oxidative stress. This study provides proof of principle that a composite score calculated from the respective weights of the biomarkers identified as highly discriminant in the statistical model (PLS) could be used as a preclinical diagnostic test to monitor biological effects of exposure to NU over time and thus in a societal context, identify low contamination effects and predict a risk of late morbidity to NU. To complete the study and improve knowledge on uranium toxicity, it would be worthwhile to identify endogenous and exogenous factors likely to increase renal sensitivity to NU and the vulnerability of individuals, such as aging, genetic factors associated with the sex, and ethnic origins. Other environmental parameters, such as pollution, disease, or drug intake, may also be considered as co-factors of exposure and aggravating factors. In further work, they must be taken into account to validate biological markers as health risk indicators usable clinically for the monitoring of nuclear workers and for other populations likely to be exposed. Indeed, metabolomics appears relevant as an analytical strategy to identify predictive markers of morbidity and to improve knowledge on and expertise in radiation protection in the field of low-dose exposure.

## Figures and Tables

**Table 1:** Clinical parameters measured in urine and plasma samples on day 5 (a) and day 270 (b) and NU concentration in kidneys measured on day 270 after exposure. The number of rats for each measurement is indicated in parentheses. Results are significantly different for: * *p* ≤ 0.05; *** *p* ≤ 0.001.

	Experimental Groups (NU Doses)	Control (20)	NU 0.5 µg/kg (20)	NU 50 µg/kg (20)	NU 500 µg/kg (20)
	**Time: Day 5**				
**(a)**	Body weight (g)	352.22 ± 4.94	363.63 ± 3.8	346.87 ± 4.78	345.75 ± 5.25
	***Urine analysis***				
	Urine volume (g/24 h)	12.76 ± 0.78	13.97 ± 0.87	13.40 ± 0.73	23.34 ± 2.47 ***
	Chlorine (mmol)	4.27 ± 0.35	4.69 ± 0.30	3.90 ± 0.34	3.40 ± 0.36
	Creatinine (µmol)	97.26 ± 3.38	100.87 ± 2.01	96.32 ± 3.07	96.24 ± 3.81
	Magnesium (mmol)	0.15 ± 0.01	0.16 ± 0.01	0.14 ± 0.02	0.27 ± 0.02 ***
	Phosphorus (mg)	0.52 ± 0.07	0.63 ± 0.06	0.57 ± 0.06	0.86 ± 0.07 ***
	Potassium (mmol)	2.94 ± 0.22	3.05 ± 0.18	2.63 ± 0.16	2.62 ± 0.18
	Sodium (mmol)	1.50 ± 0.08	1.62 ± 0.06	1.44 ± 0.08	1.48 ± 0.09
	Total proteins (mg)	0.007 ± 0.001	0.006 ± 0.001	0.006 ± 0.001	0.05 ± 0.01 ***
	Urea (mmol)	13.49 ± 0.61	14.65 ± 0.45	13.31 ± 0.60	13.30 ± 0.51
		88.83 ± 3.79	93.45 ± 3.63	95.45 ± 4.80	44.90 ± 8.09 ***
	**Time: Day 270**				
**(b)**	Uranium concentration in kidney (ng U/g)	10.15 ± 0.56	13.09 ± 1.05 *	12.84 ± 1.03 *	75.96 ± 14.64 ***
	Kidney weight (g)	1.93 ± 0.05	2.03 ± 0.06	1.86 ± 0.05	2.00 ± 0.05
	Body weight (g)	636.83 ± 10.89	653.13 ± 12.91	612.13 ± 12.84	658.00 ± 12.60
	***Urine analysis***				
	Urine volume (g/24 h)	11.60 ± 0.75	10.86 ± 0.62	11.00 ± 0.50	13.59 ± 1.30
	Albumin (mg)	4.16 ± 0.90	4.82 ± 0.84	4.12 ± 0.81	6.63 ± 1.14
	Chlorine (mmol)	2.96 ± 0.19	2.96 ± 0.16	2.70 ± 0.19	2.93 ± 0.17
	Creatinine (µmol)	115.36 ± 4.96	120.35 ± 3.31	114.88 ± 3.11	127.41 ± 3.50
	Glucose (mmol)	15.75 ± 0.86	15.57 ± 0.49	14.96 ± 0.51	18.50 ± 1.93
	Magnesium (mmol)	0.18 ± 0.01	0.18 ± 0.01	0.17 ± 0.01	0.21 ± 0.01
	Potassium (mmol)	1.57 ± 0.11	1.60 ± 0.09	1.50 ± 0.05	1.73 ± 0.10
	Sodium (mmol)	1.08 ± 0.09	1.01 ± 0.07	0.93 ± 0.05	1.00 ± 0.07
	Total proteins (mg)	58.06 ± 17.85	80.82 ± 24.73	51.38 ± 14.45	46.79 ± 10.04
	Urea (mmol)	11.53 ± 0.56	11.73 ± 0.40	11.27 ± 0.35	12.38 ± 0.49
	Uric acid (µmol)	19.39 ± 1.06	19.85 ± 0.80	19.70 ± 0.91	22.53 ± 0.86 *
	Clearance (mL/min)	1.62 ± 0.11	1.76 ± 0.11	1.75 ± 0.10	1.83 ± 0.134
	***Plasma analysis***				
	Creatinine (µmol)	48.74 ± 1.46	47.47 ± 1.57	46.09 ± 1.93	48.82 ± 1.79
	Urea (mmol)	4.71 ± 0.16	4.89 ± 0.24	4.71 ± 0.14	4.96 ± 0.13

**Table 2:** Composite score based on the nine common discriminant biomarkers for the early period (24 h to 15 days range) in urinary C18 positive profiles for reduced models 1 and 2.

Model	Individuals of Model 1 and Model 2 (24 h, 48 h, 5 d, 15 d)		Metabolite	FDR	Fold Change	Boxplot
**Validation parameters**	R2Y(cum) = 84.2% Q2(cum) = 81.9% *p* value = 5.41155 × 10^−34^	Very good permutation test	M137T39	1.1906 × 10^−10^	20.262	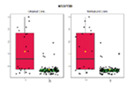
**Composite score equation**	Score = (1.10234 × 10^−10^ × M184T138) + (−5.44577 × 10^−11^ × M137T39) + (−2.31758 × 10^−9^ × M254T148) + (−1.40745 × 10^−8^ × M236T148) + (9.49838 × 10^−12^ × M153T134) + (−2.37778 × 10^−8^ × M276T148) + (2.55785 × 10^−9^ × M136T133) + (−4.47195 × 10^−9^ × M366T259) + (1.52073 × 10^−9^ × M108T133) + 0.747992		M236T148	4.0973 × 10^−21^	15.462	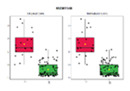
			M254T148	4.0973 × 10^−21^	14.841	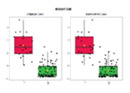
			M276T148	3.4084 × 10^−19^	11.878	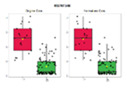
**ROC curve**	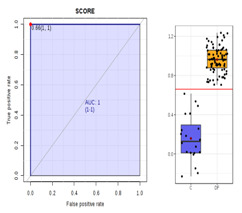	AUC = 1	M366T259	5.2434 × 10^−14^	8.2975	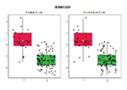
			M136T133	7.2673 × 10^−7^	0.4324	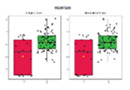
			M153T134	3.8045 × 10^−6^	0.60654	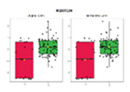
			M108T133	1.529 × 10^−5^	0.651	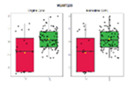
			M184T138	2.9739 × 10^−3^	0.69533	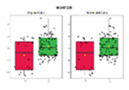

**Table 3:** The discriminant variables detected in common to at least two models calculated between the control group and the low doses of natural uranium for different sampling times in annotated urinary and plasma data.

Biological Sample/Masse (g·mol^−1^/Retention Time (s))	Primary Name	KEGG ID	CAS	HMDB/YMDB ID
***6 common discriminant variables between "M1", "M2" and "M3" (24 h to 5 days and 48 h to 5 days and 5 days to 30 days)***				
Urine_CP_M137T39	**1-Methylnicotinamide**	C02918	1005-24-9	HMDB00699
Urine_CP_M90T38	**Beta-alanine**	C00099	107-95-9	HMDB00056
Urine_CN_M221T43	**D-glucurunolactone**	C02670	32449-92-6	HMDB06355
Urine_CP_M104T39_1	***N*,*N*-dimethylglycine**	C01026	1118-68-9	HMDB0000092
Urine_CN_M209T40	**Saccharate**	C00818	576-42-1	HMDB29881
Plasma_CP_M166T208 and CP_M120T208 and CP_M149T208 or Urine_CP_M166T209 and CP_M120T209 and CP_M149T209	**L-Phenylalanine**	C00079	63-91-2	HMDB0000159
***5 common discriminant variables between "M1" and "M2" (24 h to 5 days and 48 h to 5 days)***				
Urine_CN_M145T258 and CN_M101T259	**Adipate**	C06104	124-04-9	HMDB00448
Urine_CN_M133T46 and CN_M115T46	**Malate**	C00149	97-67-6	HMDB00156
Plasma_HP_M424T124	**5b-cholanic acid-3a,12a-diol-7-one**	C04643	911-40-0	HMDB0000391
Plasma_HP_M355T137	**5b-cholanic acid-3a-ol-12-one**	No id.	5130-29-0	HMDB0000328
Plasma_CN_M475T491 and CN_M443T491 and CN_M407T491 and CN_M453T491	**Cholate**	C00695	81-25-4	HMDB00619
***3 common discriminant variables between "M1" and "M3" (24 h to 5 days and 5 days to 30 days)***				
Urine_CP_M118T53	**5-aminopentoate**	C00431	660-88-8	HMDB03355
Urine_CN_M159T299 and CN_M115T300	**6-carboxyhexnoate**	C02656	111-16-0	HMDB00857
Urine_CP_M144T297 or Plasma_CP_M144T264	**Tryptamine**	C00398	61-54-1	HMDB00303
***22 common discriminant variables between "M2" and "M3" (48 h to 5 days and 5 days to 30 days)***				
Urine_HP_M96T134	**2-Hydroxypyridine**	C02502	142-08-5	HMDB13751
Urine_HN_M165T118 and HN_M147T118	**3-(2-hydroxyphenyl propanoate**	C01198	495-78-3	HMDB33752
Urine_CN_M183T294	**3-Hydroxybenzoate**	C00587	99-06-9	HMDB02466
Urine_CP_M134T291	**5-Hydroxyindole**	No id.	1953-54-4	HMDB59805
Urine_CP_M126T46_2	**5-Methylcytosine**	C02376	58366-64-6	HMDB02894
Urine_CP_M118T40	**Betaine**	C00719	107-43-7	HMDB00043
Urine_CP_M112T40	**Cytosine**	C00380	71-30-7	HMDB00630
Urine_CP_M209T375	**dl-benzylsuccinic acid**	C09816	884-33-3	HMDB0142179
Urine_CN_M217T39_2 and CN_M227T38 and CN_M181T38	**Sorbitol**	C00749	50-70-4	HMDB00247
Urine_HP_M110T838	**Hypotaurine**	C00519	300-84-5	HMDB00965
Urine_CP_M176T374 and CP_M130T374	**Indole-3-acetate**	C00954	6505-45-9	HMDB00197
Urine_CN_M185T40	**Pentose**	No id.	No id.	No id.
Urine_CP_M166T209 and CP_M120T209 and CP_M149T209	**L-phenylalanine**	C00079	63-91-2	HMDB0000159
Urine_CP_M182T82_1	**L-Threo-3-Phenylserine (DL-3-Phenylserine)**	C03290	6254-48-4	HMDB0002184
Urine_CN_M308T40	***N*** **-acetylneuraminic acid**	C00270	131-48-6	HMDB0000230
Urine_CN_M206T343	***N*** **-acetylphenylalanine**	C03519	2018-61-3	HMDB00512
Urine_CP_M247T361	***N*** **-acetyltryptophan**	C03137	87-32-1	HMDB0013713
Urine_CP_M116T42	**Proline**	C16435	147-85-3	HMDB00162
Urine_CN_M166T56 and CN_M122T56	**Quinolinate**	C03722	89-00-9	HMDB00232
Urine_HP_M205T683 and HP_M188T684	**Tryptophan**	C00525	153-94-6	HMDB13609
Plasma_CN_M157T39	**Allantoin**	C01551	97-59-6	HMDB00462
Plasma_CP_M130T52 and CP_M84T51	**Pipecolate**	C00408	3105-95-1	HMDB00716

## Data Availability

Data are available from the corresponding authors upon request.

## Supplementary Materials

The following supporting information can be downloaded at https://www.mdpi.com/article/10.3390/metabo12050421/s1: Figure S1: PCA performed on all urine sampling times and NU doses; Figure S2: MS/MS spectra; Figure S3: PLS-DA performed on all plasma sampling times and NU dose samples; Figure S4: PCA performed on all sampling times and NU dose samples on the annotated concatenated urinary and plasma data matrices; Table S1: The characteristic and validation parameters of the four models calculated between the control group and the low doses of NU for different sampling times in urinary profiles; Table S2: New PLS-DA models calculated using the 19 common discriminant variables in at least two time-period models in urinary profiles; Table S3: The characteristic and validation parameters of the three models calculated between the control group and the low doses of NU for different sampling times in a plasma blood matrix; Table S4: PLSD-DA models calculated using the 27 discriminant variables in common to at least two time models between the control group and the low doses of NU in in plasma blood matrix; Table S5: The characteristic and validation parameters of the four models calculated between the control group and the low doses of NU for different sampling times from the annotated urinary data matrix; Table S6: The characteristic and validation parameters of the three models calculated between the control group and the low doses of NU for different sampling time in an annotated plasma matrix; Table S7: The characteristic and validation parameters of the three models calculated between the control group and the low doses of NU for different sampling time in annotated urinary and plasma data matrices; TableS8: The discriminant variables detected in common to at least two models calculated between the control group and the low doses of NU for different sampling times for annotated urinary and plasma data are available online.
