# Supplementary material for: Early Metabolomic Markers of Acute Low-Dose Exposure to Uranium in Rats
Source: Metabolites. 2022 May 7;12(5):421. doi: 10.3390/metabo12050421 (PMC9147032; doi:10.3390/metabo12050421)
Supplement: Supplementary file 1 [file metabolites-12-00421-s001.zip › metabolites-1651721-supplementary.pdf]

metabolites

MDPI

Article

Early metabolomics markers of acute low-dose exposure to uranium in rats: Supplementary Materials

Table S1. The characteristic and validation parameters of the four models calculated between the control group and the low doses of NU for different sampling times in urinary profiles. In order to identify variation in biological effects over time, four final PLS-DA models and corresponding composite scores were calculated from the urine profiles according to the treatment procedure between the control group and the low doses of NU (0.5 and 50 µg/Kg). All these models were validated in the final step after the different filtering phases and the selection of discriminating variables as summarized.
*The permutation test is considered as "very good" when the predictivity of the model decreases with the number of permutations)

	Sampling times for control/low doses of NU	PLS-DA model validation			Number of discriminant variables	Score composite validation by ROC curve
		R2Y(cum)<br>Q2(cum)	p value	Permutation test		
Model 1	24h, 48h, 5d	0.833<br>0.806	$1{,}24715 \times 10^{-25}$	Very good*	20	1
Model 2	48h, 5d, 15d	0.836<br>0.818	$8{,}16391 \times 10^{-29}$	Very good*	41	1
Model 3	5d, 15d, 30d	0.97<br>0.968	0	Very good*	33	1
Model 4	15d, 30d, 90d	0.865<br>0.777	$1{,}14004 \times 10^{-16}$	Very good*	37	0.994

Table S2. New PLS-DA models calculated using the 19 common discriminant variables in at least two time-period models in urinary profiles.

	Combining individuals from different sampling times for control/low doses of NU	PLS-DA model validation				Score composite validation by ROC curve		
		R2Y(cum)<br>Q2(cum)	Model prediction validity	p value	Permutation Test			
M′ 1	From Model 1 and Model 2	0.735<br>**0.653**	Validated	$7{,}04032 \times 10^{-22}$	Very good	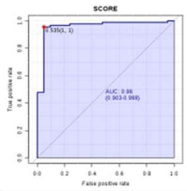 	0.957	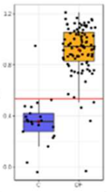
M′ 2	From M′ 1 and Model 3	0.535<br>**0.506**	Validated	$3{,}76288 \times 10^{-24}$	Very good	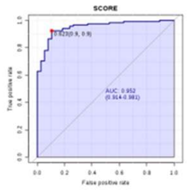 	0.951	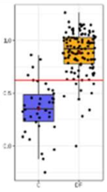
M′ 3	From M′ 2 and Model 4	**0.395**<br>**0.37**	Not validated	$1{,}16642 \times 10^{-20}$	_	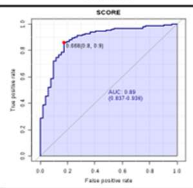 	0.890	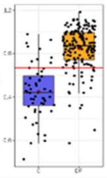

Table S3. The characteristic and validation parameters of the three models calculated between the control group and the low doses of NU for different sampling times in a plasma blood matrix.

	Sampling times for control/low doses of NU	PLS-DA model validation			Number of discriminant variables	Score composite validation by ROC curve
		R2Y(cum)<br>Q2(cum)	p value	Permutation test		
Model 1	48h, 5d, 15d	0.673<br>0. 603	$6{,}04261 \times 10^{-16}$	Very good	30	0.934
Model 2	5d, 15d, 30d	0.625<br>0.542	$3{,}36126 \times 10^{-18}$	Very good	31	0.893
Model 3	15d, 30d, 90d	0.544<br>0.52	$6{,}45441 \times 10^{-18}$	Very good	28	0.944

Table S4. PLSD-DA models calculated using the 27 discriminant variables in common to at least two time models between the control group and the low doses of NU in in plasma blood matrix.

	Combining individuals from different sampling times for control/low doses of NU	PLS-DA model validation			Score composite validation by ROC curve
		R2Y(cum)<br>Q2(cum)	p value	Permutation test	
M′ 1	From Model 1 and Model 2	0.591<br>**0.521**	$5{,}94825 \times 10^{-22}$	Very good	0.878
M′ 2	From M′ 1 and Model 3	0.322<br>**0.27**	$4{,}70312 \times 10^{-14}$	_	0.778

Table S5. The characteristic and validation parameters of the four models calculated between the control group and the low doses of NU for different sampling times from the annotated urinary data matrix. As observed for C18 positive ionization urinary data, sampling times were clearly differentiated with PCA applied to the annotated urinary data matrix (269 metabolites) and contaminated rats could not be differentiated from non-contaminated rats. In addition, there are no discriminant variables in common between the different time models. On the other hand, 40 discriminant variables in common to at least two models were selected, and new models were calculated based on these 40 variables. Unfortunately, none of the new reduced models calculated using these variables has been validated (Q2<0.5) even if the ROC curve was valid. However, there are other discriminant variables characteristic of each time period, of which the most important is tryptophan.

* metabolites associated with the nicotinate-nicotinamide pathway and ** metabolites associated with the Tryptophan pathway.

	Sampling times for control/low doses of NU	PLS-DA model validation			Score composite validation by ROC curve	Discriminant variables	
		R2Y(cum)<br>Q2(cum)	p value	Permutation Test			
Model 1	24h, 48h, 5d	0.634<br>0.536	$4{,}21649 \times 10^{-18}$	Very good	0.932	N.N-DIMETHYLGLYCINE<br>D-GLUCURONOLACTONE<br>L-GULONOLACTONE<br>4-QUINOLINECARBOXYLIC<br>7.8-DIHYDRO-L-BIOPTERIN<br>ETHYLMALONATE<br>4-PYRIDOXATE<br>**1-METHYLNICOTINAMIDE** *<br>ISO-QUINOLINE<br>ALPHA-METHYL-DL-HISTIDINE<br>DIHYDROCHLORIDE<br>L-THREO-3-PHENYLSERINE(DL-3-PHENYLSERINE)	O-HYDROXYHIPPURIC ACID<br>3-METHYLGLUTARIC ACID<br>5-AMINOPENTANOATE<br>2.6-QUINOLINEDIOL<br>(E)-3-METHYLGLUTACONIC ACID<br>NORLEUCINE<br>N-ACETYLASPARAGINE<br>HEXANOYL-L-CARNITINE<br>INOSINE<br>L-TYROSINE<br>**KYNURENIC ACID** **<br>PROLINE
Model 2	48h, 5d, 15d	0.524<br>0.502	$3{,}99664 \times 10^{-13}$	Very good	0.939	5-AMINOPENTANOATE<br>**INDOLE-3-ACETATE** **<br>L-THREO-3-PHENYLSERINE<br>NORLEUCINE<br>L-GULONOLACTONE<br>HEXANOYL-L-CARNITINE<br>2-ISOPROPYLMALIC<br>ALPHA-METHYL-DL-HISTIDINE<br>3.4-DIHYDROXY-L-PHENYLALANINE<br>**SEROTONIN** **<br>HYDROXYPHENYLLACTATE	DL-BENZYLSUCCINIC<br>N-ACETYLMETHIONINE<br>(E)-3-METHYLGLUTACONIC ACID<br>TARTRATE<br>4-METHYL-2-OXOVALERIC ACID<br>**5-HYDROXYTRYPTOPHAN** **<br>AMINOADIPATE<br>N-ACETYL-L-TYROSINE<br>3-(2-HYDROXYPHENYL)PROPANOATE<br>N-ALPHA-ACETYLLYSINE
Model 3	5d, 15d, 30d	0.635<br>0.567	$1{,}39002 \times 10^{-20}$	Very good	0.934	N.N-DIMETHYLGLYCINE<br>5-METHYLCYTOSINE<br>D-GLUCURONOLACTONE<br>3-(2-HYDROXYPHENYL)PROPANOATE<br>SACCHARATE<br>**TRYPTOPHAN** **<br>DL-BENZYLSUCCINIC<br>CYTOSINE<br>SORBITOL<br>ADIPATE<br>L-HISTIDINE<br>THIAMINE	PENTOSE<br>GLUTARATE<br>**OXOGLUTARATE** **<br>MALATE<br>BETA-ALANINE<br>**QUINOLINATE** * / **<br>HYPOTAURINE<br>3-HYDROXYBENZOATE<br>L-LYSINE<br>URATE<br>HYDROXYPHENYLLACTATE<br>1-AMINOCYCLOHEXANECARBOXYLIC
Model 4	15d, 30d, 90d	0.64<br>0.551	$2{,}13671 \times 10^{-19}$	Very good	0.959	BETA-ALANINE<br>DL-BENZYLSUCCINIC<br>L-THREO-3-PHENYLSERINE<br>3-HYDROXYBENZOATE<br>D-GLUCURONOLACTONE<br>CYTOSINE<br>3-METHYL-2-OXINDOLE<br>DELTA<br>PROLINE<br>L-GULONOLACTONE<br>3-(2-HYDROXYPHENYL)PROPANOATE<br>ALPHA-METHYL-DL-HISTIDINE<br>L-PHENYLALANINE<br>L-SERINE	HYPOTAURINE<br>**L-GLUTAMIC ACID** */**<br>L-HISTIDINE<br>N-FORMYL-L-METHIONINE<br>L-LYSINE<br>(E)-3-METHYLGLUTACONIC ACID<br>**INDOLE-3-ACETATE** **<br>**TRYPTOPHAN** **<br>SORBITOL<br>BETAINE<br>3-METHYL-2-OXOVALERATE<br>GALACTURONATE<br>5-AMINOPENTANOATE

Table S6. The characteristic and validation parameters of the three models calculated between the control group and the low doses of NU for different sampling time in an annotated plasma matrix. Similar PCA results to C18 positive ionization data were observed and the same data procedure was applied for the annotated plasma matrix where three validated models were calculated. Six discriminant metabolites were in common between all the three models with overlapping time windows and therefore can be considered as time-independent biomarkers. These metabolites were putatively identified as cholate (level 1 according to MSI), nicotinamide mononucleotide (level 3), dimeric cysteine (level 1), 5b-cholanic acid-3a-ol-12-one (level 3), 5b-cholanic acid-3a.12a-

diol-7 (level 3) and citrulline (level 1). In addition, the most interesting result from the combination of the different columns and ionization modes was the detection of new potential biomarkers such as tryptophan and nicotinamide mononucleotide, which are related to the N-methylnicotinamide pathway.

* metabolites associated with the nicotinate-nicotinamide pathway and ** metabolites associated with the Tryptophan pathway.

	Sampling times for control/low doses of NU	PLS-DA model validation			Score composite validation by ROC curve	Discriminant variables	
		R2Y(cum)<br>Q2(cum)	p value	Permutation Test			
Model 1	48h, 5d, 15d	0.61<br>0. 532	$1{,}24232 \times 10^{-15}$	Very good	0.99	L-PHENYLALANINE<br>NICOTINAMIDE MONONUCLEOTIDE<br>CYSTEINE DIMERE<br>P-OCTOPAMINE<br>2.6-DIHYDROXYBENZOIC ACID<br>L-HISTIDINE<br>CHOLATE<br>L-TYROSINE<br>GLY-PRO	5B-CHOLANIC ACID-3A-OL-12-ONE<br>L-ASPARAGINE<br>L-SERINE<br>PROLINE<br>CITRULLINE<br>2.2-DIMETHYLSUCCINIC ACID<br>5B-CHOLANIC ACID-3A.12A-DIOL-7-ONE<br>D-ORNITHINE
Model 2	5d, 15d, 30d	0.623<br>0.549	$1{,}45847 \times 10^{-17}$	Very good	0.923	5B-CHOLANIC ACID-3A-OL-12-ONE<br>CHOLATE<br>5B-CHOLANIC ACID-3A.12A-DIOL-7-ONE<br>5B-CHOLANIC ACID-3A.6A.7A-TRIOL<br>5B-CHOLANIC ACID-3A.12A-DIOL-7-ONE<br>**NICOTINAMIDE MONONUCLEOTIDE** *<br>DEOXYCHOLATE<br>5B-CHOLANIC ACID-3A-OL-12-ONE<br>5B-CHOLANIC ACID-3A-OL-7.12-DIONE<br>2-OXOADIPATE<br>GLYCOCHOLATE<br>PIPECOLATE	TRIMETHYLAMINEN-OXIDE<br>2-AMINOISOBUTYRATE<br>CITRULLINE<br>L-TYROSINE<br>5-METHYLCYTOSINE<br>**OXOGLUTARATE** **<br>CYSTEINE DIMERE<br>MEVALONOLACTONE HYDRATE<br>P-OCTOPAMINE<br>(S)-3-HYDROXYBUTYRIC ACID -H- ET (S)-3-HYDROXYBUTYRIC ACID<br>16-0 LYSOPI
Model 3	15d, 30d, 90d	0.596<br>0.518	$9{,}70734 \times 10^{-16}$	Very good	0.95	NICOTINAMIDE MONONUCLEOTIDE<br>5B-CHOLANIC ACID-3A-OL-12-ONE<br>5B-CHOLANIC ACID-3A.12A-DIOL-7-ONE<br>CHOLATE<br>18-0 LYSOPC<br>CITRULLINE<br>5B-CHOLANIC ACID-3A.6A.7A-TRIOL<br>DEOXYCHOLATE<br>16-0 LYSOPC	ALLANTOIN<br>**5-HYDROXYINDOLE-3-ACETIC-ACID** **<br>2.6-QUINOLINEDIOL<br>**TRYPTOPHAN** **<br>C16 PLASMLPC<br>CYSTEINE DIMERE<br>C18 LYSOPAF<br>18-0 LYSOPE<br>D-ORNITHINE

Table S7. The characteristic and validation parameters of the three models calculated between the control group and the low doses of NU for different sampling time in annotated urinary and plasma data matrices.

	Sampling times for control/low doses of NU	PLS-DA model validation			Number of discriminant variables	Score composite validation by ROC curve
		R2Y(cum) Q2(cum)	p value	Permutation test		
Model 1	48h, 5d, 15d	0.714 0.597	4,02985 × 10^{-21}	Very good	52	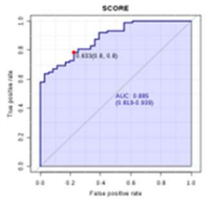 0.884 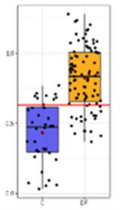
Model 2	5d, 15d, 30d	0.791 0.64	6,56191 × 10^{-26}	Very good	49	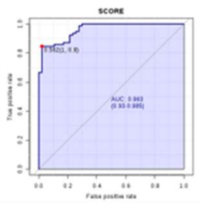 0.961 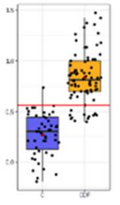
Model 3	15d, 30d, 90d	0.678 0.57	6,82626 × 10^{-24}	Very good	53	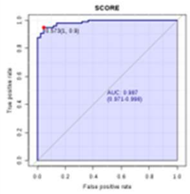 0.986 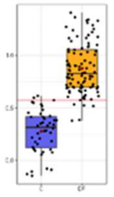

Tables S8 a, b, c, d. The discriminant variables detected in common to at least two models calculated between the control group and the low doses of NU for different sampling times for annotated urinary and plasma data.* Model calculated between control samples and low dose NU for different sampling times between 48h, 5d and 15d; 5d, 15d and 30d; 15d, 30d and 90d.

		48 hours to 15 days				5 days to 30 days				15 days to 90 days			
a). 6 common discriminant variables between 48 hours to 15 days, 5 days to 30 days and 15 days to 90 days		Fold Change	p.value	FDR	Box plot	Fold Change	p.value	FDR	Box plot	Fold Change	p.value	FDR	Box plot
Urine_CP_M137T39	1-METHYLNICOTINAMIDE_+H+	4,5475	$5,93 \times 10^{-05}$	$1,34 \times 10^{-04}$		3,0335	$1,30 \times 10^{-02}$	$1,30 \times 10^{-02}$		3,3398	$1,99 \times 10^{-03}$	$5,27 \times 10^{-03}$	
Urine_CP_M90T38	BETA-ALANINE_+H+	0,35182	$1,28 \times 10^{-05}$	$4,76 \times 10^{-05}$		0,41475	$4,53 \times 10^{-05}$	$2,78 \times 10^{-04}$		0,22336	$1,94 \times 10^{-05}$	$5,14 \times 10^{-04}$	
Urine_CN_M221T43	D-GLUCURONOLACTONE_+HCOOH-H-	0,42357	$3,11 \times 10^{-02}$	$3,59 \times 10^{-02}$		0,16749	$3,98 \times 10^{-06}$	$4,87 \times 10^{-05}$		0,38612	$1,74 \times 10^{-03}$	$4,87 \times 10^{-03}$	
Urine_CP_M104T39_1	N.N-dimethylglycine_+H+	0,34799	$1,27 \times 10^{-04}$	$2,64 \times 10^{-04}$		0,18562	$1,40 \times 10^{-05}$	$9,80 \times 10^{-05}$		0,2196	$2,21 \times 10^{-03}$	$5,27 \times 10^{-03}$	
Urine_CN_M209T40	SACCHARATE_-H-	0,1107	$2,26 \times 10^{-06}$	$1,96 \times 10^{-05}$		0,40602	$8,08 \times 10^{-05}$	$3,96 \times 10^{-04}$		0,42062	$4,11 \times 10^{-04}$	$2,18 \times 10^{-03}$	
Plasma_CP_M166T208 and CP_M120T208 and CP_M149T208 or Urine_CP_M166T209 and CP_M120T209 and CP_M149T209	L-Phenylalanine__+H+ and L-Phenylalanine__-HCOOH+H+ and L-Phenylalanine__-NH3+H+	0,58505	$3,19 \times 10^{-04}$	$1,29 \times 10^{-03}$		0,37348	$2,07 \times 10^{-03}$	$4,39 \times 10^{-03}$		0,23952	$1,95 \times 10^{-04}$	$1,44 \times 10^{-03}$	

		48 hours to 15 days				5 days to 30 days				15 days to 90 days			
b). 5 common discriminant variables between	48 hours to 15 days and 5 days to 30 days	Fold Change	p.value	FDR	Box plot	Fold Change	p.value	FDR	Box plot	Fold Change	p.value	FDR	Box plot
Urine_CN_M145T258 and CN_M101T259	ADIPATE_-H- and ADIPATE_-H-CO2	2,6988	$4,24 \times 10^{-05}$	$1,05 \times 10^{-04}$		2,1559	$6,23 \times 10^{-03}$	$7,63 \times 10^{-03}$					
Urine_CN_M133T46 and CN_M115T46	MALATE_-H- and MALATE_-H2O-H-	2,623	$4,13 \times 10^{-05}$	$1,05 \times 10^{-04}$		2,2175	$2,57 \times 10^{-03}$	$4,80 \times 10^{-03}$					
Plasma_HP_M424T124	5b-cholanic acid-3a.12a-DIOL-7-ONE_+NH4+	0,4261	$2,20 \times 10^{-02}$	$2,66 \times 10^{-02}$		0,34482	$1,87 \times 10^{-03}$	$4,28 \times 10^{-03}$					
Plasma_HP_M355T137	5b-cholanic acid-3a-OL-12-ONE_-2H2O+H+	0,51625	$2,70 \times 10^{-02}$	$3,19 \times 10^{-02}$		0,42487	$1,23 \times 10^{-03}$	$3,02 \times 10^{-03}$					
Plasma_CN_M475T491 and CN_M443T491 and CN_M407T491 and CN_M453T491	CHOLATE_+NaHCO2-H- and CHOLATE_+Cl- and CHOLATE_+H- and CHOLATE_+HCOOH-H-	0,46858	$1,07 \times 10^{-02}$	$1,43 \times 10^{-02}$		0,46869	$4,28 \times 10^{-03}$	$6,77 \times 10^{-03}$					

		48 hours to 15 days				5 days to 30 days				15 days to 90 days			
c). 3 common discriminant variables bandween	48 hours to 15 days and 15 days to 90 days	Fold Change	p.value	FDR	Box plot	Fold Change	p.value	FDR	Box plot	Fold Change	p.value	FDR	Box plot
Urine_CP_M118T53	5-AMINOPENTANOATE_+H+	0,28797	$5,58 \times 10^{-01}$	$5,69 \times 10^{-01}$						0,25579	$9,29 \times 10^{-03}$	$1,27 \times 10^{-02}$	
Urine_CN_M159T299 and CN_M115T300	6-CARBOXYHEXANOATE_-H- and 6-CARBOXYHEXANOATE_-H-CO2	2,2881	$1,63 \times 10^{-04}$	$3,26 \times 10^{-04}$						1,7233	$8,38 \times 10^{-03}$	$1,27 \times 10^{-02}$	
Urine_CP_M144T297 or Plasma_CP_M144T264	TRYPTAMINE_-NH3+H+	1,4268	$2,02 \times 10^{-02}$	$2,50 \times 10^{-02}$						0,45513	$1,30 \times 10^{-02}$	$1,44 \times 10^{-02}$	

		48 hours to 15 days				5 days to 30 days				15 days to 90 days			
d). 22 common discriminant variables bandween	5 days to 30 days and 15 days to 90 days	Fold Change	p.value	FDR	Box plot	Fold Change	p.value	FDR	Box plot	Fold Change	p.value	FDR	Box plot
Urine_HP_M96T134	2-HYDROXYPYRIDINE_+H+					2,5202	$2,15 \times 10^{-03}$	$4,39 \times 10^{-03}$		2,4274	$2,23 \times 10^{-03}$	$5,27 \times 10^{-03}$	
Urine_HN_M165T118 and HN_M147T118	3-(2-HYDROXYPHENYL)PROPANOATE_-H- and 3-(2-HYDROXYPHENYL)PROPANOATE_-H2O-H-					3,2462	$5,75 \times 10^{-06}$	$5,64 \times 10^{-05}$		2,3569	$2,29 \times 10^{-03}$	$5,27 \times 10^{-03}$	
Urine_CN_M183T294	3-HYDROXYBENZOATE_+HCOOH-H-					0,16846	$4,88 \times 10^{-04}$	$1,49 \times 10^{-03}$		0,15749	$1,54 \times 10^{-04}$	$1,43 \times 10^{-03}$	
Urine_CP_M134T291	5-Hydroxyindole_+H+					3,4989	$1,08 \times 10^{-03}$	$2,94 \times 10^{-03}$		2,8697	$9,91 \times 10^{-03}$	$1,27 \times 10^{-02}$	
Urine_CP_M126T46_2	5-METHYLCYTOSINE_+H+					3,0805	$1,30 \times 10^{-06}$	$3,17 \times 10^{-05}$		2,2542	$5,65 \times 10^{-04}$	$2,71 \times 10^{-03}$	
Urine_CP_M118T40	BETAINE_+H+					0,17949	$2,65 \times 10^{-03}$	$4,80 \times 10^{-03}$		0,25579	$1,15 \times 10^{-03}$	$3,80 \times 10^{-03}$	
Urine_CP_M112T40	CYTOSINE_+H+					3,4712	$2,63 \times 10^{-06}$	$4,29 \times 10^{-05}$		2,6671	$3,96 \times 10^{-05}$	$7,00 \times 10^{-04}$	
Urine_CP_M209T375	dl-benzylsuccinic_acid_+H+					0,17403	$1,23 \times 10^{-05}$	$9,80 \times 10^{-05}$		0,29352	$1,02 \times 10^{-04}$	$1,35 \times 10^{-03}$	
Urine_CN_M217T39_2 and CN_M227T38 and CN_M181T38	SORBITOL_+Cl- and SORBITOL_+HCOOH-H- and SORBITOL_-H-					3,9214	$7,39 \times 10^{-07}$	$3,17 \times 10^{-05}$		2,487	$6,45 \times 10^{-04}$	$2,71 \times 10^{-03}$	
Urine_HP_M110T838	HYPOTAURINE_+H+					0,26261	$2,89 \times 10^{-04}$	$1,10 \times 10^{-03}$		0,27312	$2,63 \times 10^{-03}$	$5,80 \times 10^{-03}$	
Urine_CP_M176T374 and CP_M130T374	INDOLE-3-ACETATE_+H+ and INDOLE-3-ACETATE_-HCOOH+H+					0,2941	$1,21 \times 10^{-03}$	$3,02 \times 10^{-03}$		0,20942	$1,62 \times 10^{-04}$	$1,43 \times 10^{-03}$	
Urine_CN_M185T40	Pentose_1					2,4023	$2,91 \times 10^{-04}$	$1,10 \times 10^{-03}$		1,6262	$8,76 \times 10^{-03}$	$1,27 \times 10^{-02}$	
Urine_CP_M166T209 and CP_M120T209 and CP_M149T209	L-Phenylalanine__+H+ and L-Phenylalanine__-HCOOH+H+ and L-Phenylalanine__-NH3+H+					0,37348	$2,07 \times 10^{-03}$	$4,39 \times 10^{-03}$		0,23952	$1,95 \times 10^{-04}$	$1,44 \times 10^{-03}$	
Urine_CP_M182T82_1	L-Threo-3-Phenylserine_(DL-3-Phenylserine)_+H+					0,35991	$7,66 \times 10^{-03}$	$8,93 \times 10^{-03}$		0,14457	$2,45 \times 10^{-04}$	$1,44 \times 10^{-03}$	
Urine_CN_M308T40	N-Acetylneuraminic_acid_-H					0,3367	$4,65 \times 10^{-03}$	$6,91 \times 10^{-03}$		0,27138	$7,67 \times 10^{-04}$	$2,71 \times 10^{-03}$	
Urine_CN_M206T343	N-ACETYLPHENYLALANINE_-H-					3,6120	$5,93 \times 10^{-03}$	$7,52 \times 10^{-03}$		3,3627	$1,03 \times 10^{-02}$	$1,27 \times 10^{-02}$	
Urine_CP_M247T361	N-ACETYLTRYPTOPHAN_+H+					2,0207	$5,64 \times 10^{-03}$	$7,52 \times 10^{-03}$		1,6865	$1,04 \times 10^{-02}$	$1,27 \times 10^{-02}$	
Urine_CP_M116T42	PROLINE_+H+					0,31267	$4,12 \times 10^{-04}$	$1,35 \times 10^{-03}$		0,16027	$9,06 \times 10^{-07}$	$4,80 \times 10^{-05}$	
Urine_CN_M166T56 and CN_M122T56	QUINOLINATE_-H- and QUINOLINATE_-H-CO2					0,42288	$5,63 \times 10^{-03}$	$7,52 \times 10^{-03}$		0,41909	$2,80 \times 10^{-03}$	$5,90 \times 10^{-03}$	
Urine_HP_M205T683 and HP_M188T684	TRYPTOPHAN_+H+ and TRYPTOPHAN_-NH3+H+					0,22264	$5,36 \times 10^{-05}$	$2,92 \times 10^{-04}$		0,4117	$9,62 \times 10^{-03}$	$1,27 \times 10^{-02}$	
Plasma_CN_M157T39	ALLANTOIN_-H-					1,9932	$5,98 \times 10^{-03}$	$7,52 \times 10^{-03}$		2,5354	$1,22 \times 10^{-03}$	$3,80 \times 10^{-03}$	
Plasma_CP_M130T52 and CP_M84T51	PIPECOLATE_+H+ and PIPECOLATE_-HCOOH+H+					2,0969	$3,73 \times 10^{-04}$	$1,30 \times 10^{-03}$		1,5738	$7,86 \times 10^{-03}$	$1,27 \times 10^{-02}$	

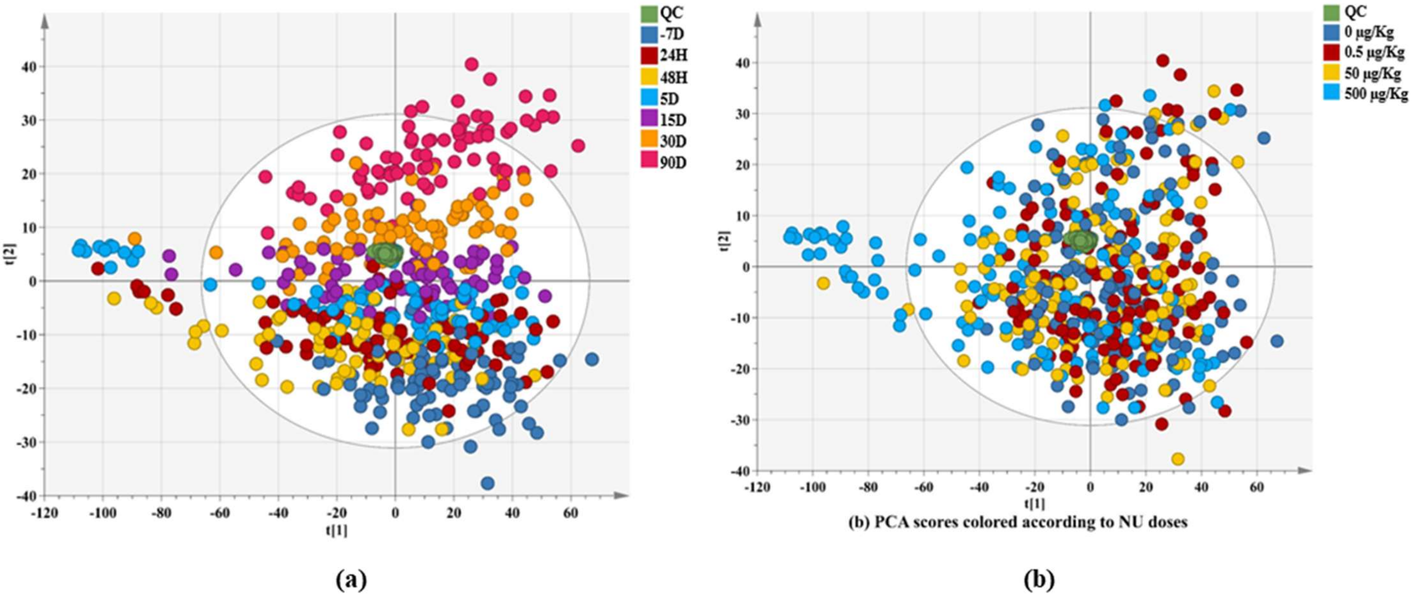

Figure S1. PCA performed on all urine sampling times and NU doses. PCA is a descriptive analysis method used to visualize the distribution of samples in a low dimensional space and demonstrate a possible structuring in the data without any information on the nature of samples. **Figure S1a**,**b** are the same but just colored differently according to **(a)** sampling time regardless of uranium dose groups and **(b)** to NU doses regardless of the time collection point without considering this information during the calculation of PCA. In **(a)**, the sampling time effect is visible on the second PCA component.

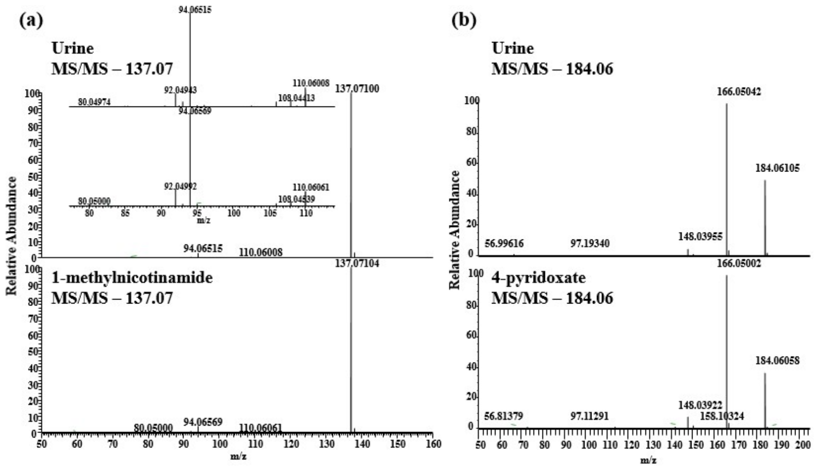

Figure S2. MS/MS spectra of the precursor $[M + H]^+$ ions at (**a**) m/z 137 putatively identified as 1-methylnicotinamide and (**b**) m/z 184 putatively identified as 4-pyridoxate from the authentic standard and from the pool urine sample.

PLS-DA performed on all plasma sampling times and NU doses

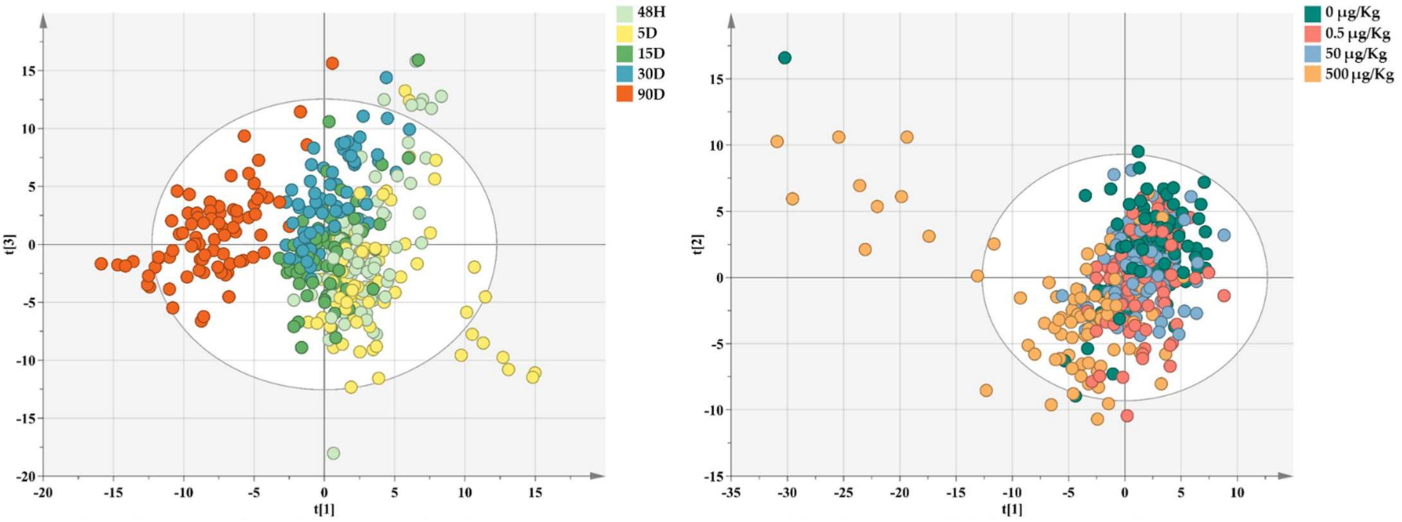

(a) PLS-DA scores with sampling times as predictor Y variable (b) PLS-DA scores with NU doses as predictor Y variable

Figure S3. PLS-DA performed on all plasma sampling times and NU dose samples. **(a)** Score plot with sampling times as the predictor Y variable. **(b)** Score plot with NU doses as the predictor Y variable. PCA is a descriptive analysis method used to visualize the distribution of samples in a low dimensional space and demonstrate a possible structuring in the data without any information on the nature of samples. **Figure S2a**,**b** are the same but just colored differently according to **(a)** sampling time regardless of uranium dose groups and **(b)** to NU doses regardless of the time collection point without considering this information during the calculation of PCA.

PCA performed on the annotated concatenated urinary and plasma data matrices

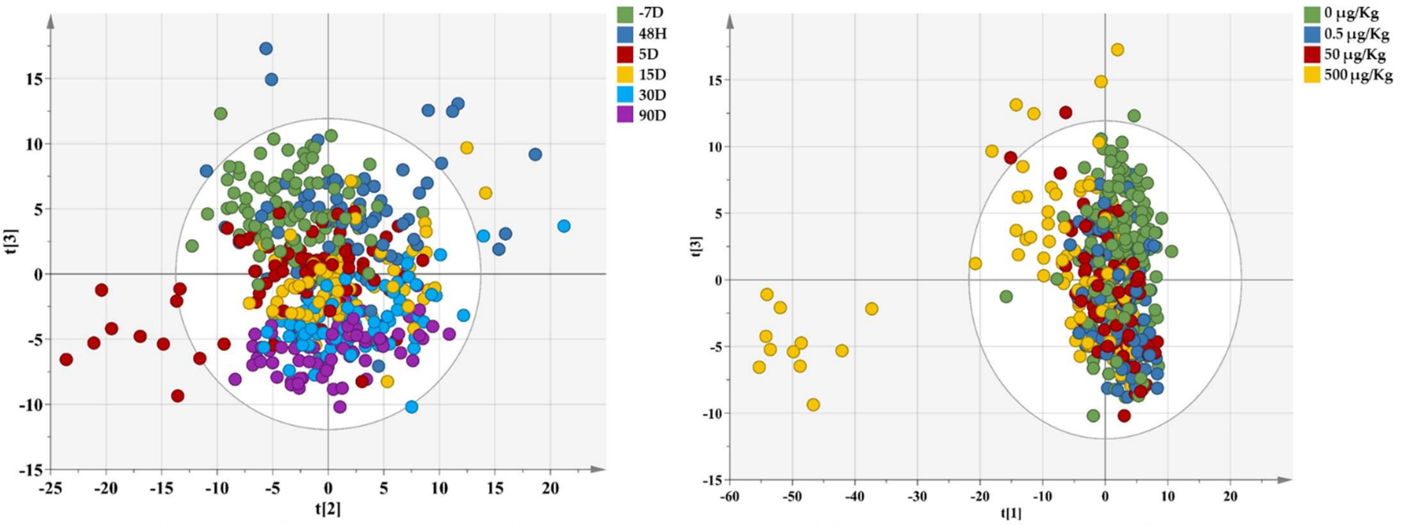

(a) PCA scores plot colored according to sampling times (b) PCA scores plot colored according to NU doses

Figure S4.PCA performed on all sampling times and NU dose samples on the annotated concatenated urinary and plasma data matrices **(a)** Score plot colored according to sampling time. **(b)** Score plot colored according to NU dose. **Figure S3a**,**b** are the same but just colored differently according to **(a)** sampling time regardless of uranium dose groups and **(b)** to NU doses regardless of the time collection point without considering this information during the calculation of PCA.
